# Supplementary material for: A metabolism-chromatin axis promotes differential ribosomal RNA transcription in the human malaria parasite
Source: Nat Commun. 2025 Dec 19;17:818. doi: 10.1038/s41467-025-67522-w (PMC12824157; doi:10.1038/s41467-025-67522-w)
Supplement: Supplementary file 2 — Description of Additional Supplementary Files [file 41467_2025_67522_MOESM2_ESM.pdf]

Description of additional supplementary files

Supplementary Data 1: *P. falciparum* rDNA annotation
Annotation and sequence comparison of the seven *P. falciparum* rDNA loci described in this manuscript.

Supplementary Data 2: The PolI machinery of *P. falciparum*
Results of the search for homologous proteins related to PolI activity in *P. falciparum.*
Sheet 1: GO terms used to retrieve model organism protein sequences related to PolI activity.
Sheet 2: BLASTp results and annotation

Supplementary Data 3: HMGB1/2 ChIP analysis
Results of peak calling for HMGB1 and HMGB2 chromatin immunoprecipitation (ChIP-seq) experiments. *p*-values and *q*-values (i.e. FDR-adjusted *p*-values) were calculated in macs2.

Sheet 1: Significant peaks in HMGB1 ChIP-seq of P. falciparum grown at 37°C
Sheet 2: Significant peaks in HMGB1 ChIP-seq of P. falciparum grown at 32°C
Sheet 3: Significant peaks in HMGB2 ChIP-seq of P. falciparum grown at 37°C

Supplementary Data 4: rRNA expression analysis
Normalized expression values in FPKM (Fragments per kilobase of exon per one million mapped reads) of the different 28S rRNA-types for all experiments presented in this manuscript

Supplementary Data 5: Differental HMGB1 peak analysis
Detection of differentially enriched peaks in HMGB1 ChIP-seq experiments of parasites grown at 37°C and 32°C.

Supplementary Data 6: Temperature-dependent *P. facliparum* gene expression
Results of the differential gene expression analyis of parasites grown at 37°C and 32°C. *p*-values and the FDR-adjusted *p*-values were calculated in DESeq2.

Supplementary Data 7: Targeted LC-MS metabolomics analysis
Results of quantitative metabolomics. Values are area under the curve (auc) per 1x10^6 cells

Supplementary Data 8: Histone LC-MS/MS results
Histone post-translational modifications identified on histone H3 (PF3D7_0610400) and H4 (PF3D7_1105000) of wildtype (strain 3D7) and Sir2a-KO *P. falciparum* parasites
Sheet 1: Summary of proteotypic peptids detected in H3 and H4
Sheet 2: Normalized log2-transformed intensities of each proteotypic H3 and H4 peptide
Sheet 3: Z-scores of normalized and log2-transformed intensities of each individual peptide
Sheet 4: Aggregated Z-scores for individual histone PTM combinations
Note: Carboxyethylation and lactylation PTMs have an identical mass and cannot unequivocally be distinguished
p-values were calculated using a two-sided *t*-test and adjusted using the Benjamini-Hochberg method.

Supplementary Data 9: Differential madID analysis
Enriched regions (FC >=10) between the Sir2a-madID and control madID cell line

Supplementary Data 10: Histone PTM ChIP analysis
Results of peak calling for H3K9ac, H3K14ac, H4K12ac and H4K16ac ChIP-seq experiments in wild-type (strain 3D7) and P. falciparum Sir2a-KO parasites.

Info: For each sample, overlapping peaks of two replicates are shown. The results represent the significant peaks identified by comparing ChIP over input signal. The normalized enrichment in counts per million (CPM, calibrated to the yeast spike in) at the peak summit position is also represented for each sample and replicate.
p-values and *q*-values (i.e. FDR-adjusted *p*-values) were calculated in macs2

Supplementary Data 11: 6mA analysis at rDNA loci
6mA enrichments in rDNA upstream regions

Supplementary Data 12: Oligos and primer list
Primers used for plasmid cloning and verification of cell lines

Supplementary Data 13: *P. falciparum* growth measurement
Growth phenotyping of *P. falciparum* strains described in this manuscript

Supplementary Data 14: Raw data accessions
Accession numbers at the NCBI Sequence Read Archive
