# Supplementary material for: A metabolism-chromatin axis promotes differential ribosomal RNA transcription in the human malaria parasite
Source: Nat Commun. 2025 Dec 19;17:818. doi: 10.1038/s41467-025-67522-w (PMC12824157; doi:10.1038/s41467-025-67522-w)
Supplement: Supplementary file 17 — Reporting summary [file 41467_2025_67522_MOESM17_ESM.pdf]

nature portfolio

Corresponding author(s): Sebastian Baumgarten

Last updated by author(s): Nov 27, 2025

Reporting Summary

Nature Portfolio wishes to improve the reproducibility of the work that we publish. This form provides structure for consistency and transparency in reporting. For further information on Nature Portfolio policies, see our Editorial Policies and the Editorial Policy Checklist.

Statistics

For all statistical analyses, confirm that the following items are present in the figure legend, table legend, main text, or Methods section.

n/a	Confirmed	
☐	☒	The exact sample size (*n*) for each experimental group/condition, given as a discrete number and unit of measurement
☐	☒	A statement on whether measurements were taken from distinct samples or whether the same sample was measured repeatedly
☐	☒	The statistical test(s) used AND whether they are one- or two-sided<br>*Only common tests should be described solely by name; describe more complex techniques in the Methods section.*
☒	☐	A description of all covariates tested
☒	☐	A description of any assumptions or corrections, such as tests of normality and adjustment for multiple comparisons
☐	☒	A full description of the statistical parameters including central tendency (e.g. means) or other basic estimates (e.g. regression coefficient) AND variation (e.g. standard deviation) or associated estimates of uncertainty (e.g. confidence intervals)
☐	☒	For null hypothesis testing, the test statistic (e.g. *F*, *t*, *r*) with confidence intervals, effect sizes, degrees of freedom and *P* value noted<br>*Give P values as exact values whenever suitable.*
☒	☐	For Bayesian analysis, information on the choice of priors and Markov chain Monte Carlo settings
☒	☐	For hierarchical and complex designs, identification of the appropriate level for tests and full reporting of outcomes
☐	☒	Estimates of effect sizes (e.g. Cohen's *d*, Pearson's *r*), indicating how they were calculated

Our web collection on statistics for biologists contains articles on many of the points above.

Software and code

Policy information about availability of computer code

Data collection	BioRad Image Lab Sofware 5.2<br>Zeiss ZEN 2012 SP5 FP3 (black) v14.0<br>AB SCIEX Analyst v1.7.2<br>AB SCIEX OS v2.0.0<br>Thermo Scientific Xcalibur v4.0-4.0262.18-SP1/4.0.309.28
Data analysis	hicExplorer v3.7.2<br>juicebox v2.15<br>bcl2fastq v2.20.0.422<br>trimmomatic v0.39<br>bowtie2 v2.5.1<br>macs2 v2.2.9.1<br>tinymapper v0.14.1<br>STAR v2.7.11b<br>htseq-count v2.0.3<br>bedtools v2.31.0<br>samtools v1.15.1<br>bedops v2.4.41<br>deeptools v3.5.1<br>minimap2 v2.26-r1175<br>dorado v0.8.2

Mascot v2.5.1
uncalled4 v4.1.0
pod5 v0.3.15
blastp v2.14.0
Fiji v2.9.0
Skyline, v23.1.0.380
myProMS, v3.10
R v4.4.1
RStudio v2024.04.2+764

For manuscripts utilizing custom algorithms or software that are central to the research but not yet described in published literature, software must be made available to editors and reviewers. We strongly encourage code deposition in a community repository (e.g. GitHub). See the Nature Portfolio guidelines for submitting code & software for further information.

Data

Policy information about availability of data

All manuscripts must include a data availability statement. This statement should provide the following information, where applicable:

- Accession codes, unique identifiers, or web links for publicly available datasets
- A description of any restrictions on data availability
- For clinical datasets or third party data, please ensure that the statement adheres to our policy

All raw Illumina sequencing reads and aligned ONT samples are available at NCBI BioProject with accession PRJNA1224397. An overview Table of all samples with the respective SRA number is shown in Supplementary Table 13. ChIP-seq and madID tracks are deposited at the Gene Expression Omnibus (GEO) database with accession GSE290936 (madID) and GSE290639 (ChIP-seq). The mass spectrometry proteomics data have been deposited to the ProteomeXchange Consortium (http://proteomecentral.proteomexchange.org) via the PRIDE partner repository94 with the dataset identifier PXD060142.
Previously published data used in this manuscript are available under the following accession numbers:
HP1 (Carrington et al., 2021) ChIP: SRR12281320
HP1 (Carrington et al., 2021) Input: SRR12281322
ATAC (Toenhake et al., 2018): SRR6055333
ATAC (Toenhake et al, 2018) gDNA control: SRR6055335
MicroC (Singh et al., 2025): GSE278141

Research involving human participants, their data, or biological material

Policy information about studies with human participants or human data. See also policy information about sex, gender (identity/presentation), and sexual orientation and race, ethnicity and racism.

Reporting on sex and gender	NA. Red blood cells were donated to the Etablissement Francais du Sang and fully anonymized.
Reporting on race, ethnicity, or other socially relevant groupings	NA. Red blood cells were donated to the Etablissement Francais du Sang and fully anonymized.
Population characteristics	NA. Red blood cells were donated to the Etablissement Francais du Sang and fully anonymized.
Recruitment	The red blood cells were donated to, and purchased from the Etablissement Francais du Sang.
Ethics oversight	The red blood cells were purchased from the Etablissement Francais du Sang with approval number HS 2021-24819.

Note that full information on the approval of the study protocol must also be provided in the manuscript.

Field-specific reporting

Please select the one below that is the best fit for your research. If you are not sure, read the appropriate sections before making your selection.

☒ Life sciences ☐ Behavioural & social sciences ☐ Ecological, evolutionary & environmental sciences

For a reference copy of the document with all sections, see nature.com/documents/nr-reporting-summary-flat.pdf

Life sciences study design

All studies must disclose on these points even when the disclosure is negative.

Sample size	Sample sizes for RNA- and ChIP-seq experiments were determined as recommended by the ENCODE standards. Histone mass-spectrometry and metabolomic sample sizes were determined according to the minimal requirements for statistical analyses. Input material was used in quantities according to experiences from previous studies (e.g. PMID 40588593).
Data exclusions	No data were excluded from the study.
Replication	All RNA-seq experiments were performed in triplicates. ChIP-seq exeperiments were performed in two biologically independent duplicates.

Replication	For histone mass-spectrometry, six biological replicates were performed per condition. For targeted metabolomics, between 5 and 9 biological replicates were performed for each condition tested.
Randomization	Our study did not require randomization. Covariates are not relevant for this study.
Blinding	Our study did not require blinding. It did not involve human or animal cohorts or subjective counting.

Reporting for specific materials, systems and methods

We require information from authors about some types of materials, experimental systems and methods used in many studies. Here, indicate whether each material, system or method listed is relevant to your study. If you are not sure if a list item applies to your research, read the appropriate section before selecting a response.

Materials & experimental systems

n/a	Involved in the study
☐	☒ Antibodies
☐	☒ Eukaryotic cell lines
☒	☐ Palaeontology and archaeology
☒	☐ Animals and other organisms
☒	☐ Clinical data
☒	☐ Dual use research of concern
☒	☐ Plants

Methods

n/a	Involved in the study
☐	☒ ChIP-seq
☐	☒ Flow cytometry
☒	☐ MRI-based neuroimaging

Antibodies

Antibodies used	For Western Blots, Histone H3 was detected with anti-H3 (Abcam # ab1791: 1:1,000 in 1% milk-PBST) primary antibody, followed by donkey anti-rabbit (GE # NA934-1ML) secondary antibody conjugated to HRP (1:5,000). HA-tagged proteins and PfAldolase were detected using HRP conjugated anti-HA (Ozyme 14031S, 1:1,000 in 1% milk PBST), and anti-PfAldolase (Abcam # ab38905, 1:5,000 in 1% milk PBST) antibodies, respectively. All ChiP-seq experiments were performed using a ChIP-grade anti-HA antibody (Abcam #ab9110). IFA experiments were performed with anti-HA clone 3F10 (Sigma 12158167001).
Validation	All primary antibodies (Abcam: ab9110, ab1791 and ab38905; Ozyme: 14031S; Sigma: 12158167001) are guaranteed by the manufacturers for use in the corresponding experiment (i.e. Western Blot, IFA and ChIP). We have also validated these antibodies in previous studies (e.g. PMID: 40588593).

Eukaryotic cell lines

Policy information about cell lines and Sex and Gender in Research

Cell line source(s)	The wildtype cell lines used are clones of the strains NF54 or 3D7. The Sir2 knockout cell line was first described in PMID: 15820675. All cell lines used in this study were based on a clone of the NF54 wild-type cell line.
Authentication	We used PCR followed by Sanger sequencing or direct DNA Nanopore sequencing to confirm the correct integration of all epitope tags and glmS ribozyme sequences a the targeted endogenous loci.
Mycoplasma contamination	All strains have tested negative for mycoplasma
Commonly misidentified lines (See ICLAC register)	No commonly misidentified lines were used in this study.

Plants

Seed stocks	NA
Novel plant genotypes	NA
Authentication	NA

ChIP-seq

Data deposition

☒ Confirm that both raw and final processed data have been deposited in a public database such as GEO.

☒ Confirm that you have deposited or provided access to graph files (e.g. BED files) for the called peaks.

Data access links<br>*May remain private before publication.*	All raw Illumina sequencing reads and aligned ONT samples are available at NCBI BioProject with accession PRJNA1224397. An overview Table of all samples with the respective SRA number is shown in Supplementary Table 13. ChIP-seq and madID tracks are deposited at the Gene Expression Omnibus (GEO) database with accession GSE290936 (madID) and GSE290639 (ChIP-seq).
Files in database submission	Please see Supplementary Table 14
Genome browser session<br>(e.g. UCSC)	NA

Methodology

Replicates	All ChIP-seq experiments were performed in two biological replicates. Overlap of significant peaks identified by macs2 were determined using bedtools 'intersect'.
Sequencing depth	Please see Supplementary Table 14
Antibodies	All proteins used in ChIP-seq experiments were tagged with a 3xHA tag and immunoprecipitated with a ChIP-grade anti-HA antibody (Abcam #ab9110).
Peak calling parameters	Raw read pre-processing and adapter trimming were performed using bcl2fastq and trimmomatic. Trimmed reads were aligned to the masked genome using bowtie2 with settings '--no-mixed --no-discordant --end-to-end --sensitive'. Alignments were subsequently filtered for PCR duplicates using samtools84 'fixmate' and 'markdup' and only alignments with a mapping quality ≥ 30 were retained (samtools view -q 30). To calculate differences in HMGB1 occupancy at rDNA loci at 32°C and 37°C, IP/input enrichments were first calculated for both conditions and replicates independently using macs2 'callpeak' with options '--nomodel --extsize 120'. The resulting tag numbers, pileup and lambda files for the IP and input samples of each replicate and condition were then used to calculate significant enrichment differences using macs2 'bdgdiff '. For all ChIP-seq experiments, coverage tracks representing the ratio of IP over Input were generated using deeptool's 'bamCompare' with options '-bs 10 --scaleFactorsMethod None --operation ratio --normalizeUsing CPM'. For the analysis of histone PTMs in the wild-type-3D7 and Sir2a-KO parasites, raw sequencing reads were pre-processed as described above. The reads were then mapped to the masked P. falciparum genome (PlasmoDB v64) using tinymapper (https://github.com/js2264/tinyMapper), with option '--mode ChIP' and using the S. cerevisiae S288C genome (version R64) as calibration reference. Only correctly paired reads with a mapping quality ≥ 30 were retained (bowtie2 option '-f 0x001 -f 0x002' -q 30) for downstream analysis within tinymapper. Significant peaks for each replicate and histone PTM were identified within tinymapper using macs2 and by using the input sample as background. To calculate quantitative enrichment values for each PTM, for each sample and replicate, the number of ChIP reads mapping to the P. falciparum genome (in counts per million [CPM]) was normalized to the total number of ChIP reads mapping to the S. cerevisiae calibration genome (Supplementary Table 10), allowing for the normalization of absolute peak enrichment (i.e. peak height) between samples (Supplementary Fig. 7e). Overlapping consensus peaks between replicates were identified using bedtool's 'intersect' (Supplementary Table 10).
Data quality	All peaks had a adjusted q-value < 0.05. The overlap of peaks between the two independent replicates ranges from 77-99%.
Software	bcl2fastq v2.20.0.422<br>trimmomatic v0.39<br>bowtie2 v2.5.1<br>macs2 v2.2.9.1<br>tinymapper v0.14.1<br>bedtools v2.31.0<br>samtools v1.15.1<br>deeptools v3.5.1

Flow Cytometry

Plots

Confirm that:

☒ The axis labels state the marker and fluorochrome used (e.g. CD4-FITC).

☒ The axis scales are clearly visible. Include numbers along axes only for bottom left plot of group (a 'group' is an analysis of identical markers).

☒ All plots are contour plots with outliers or pseudocolor plots.

☒ A numerical value for number of cells or percentage (with statistics) is provided.

Methodology

Sample preparation	To measure parasite growth kinetics the cell lines were tightly synchronized by plasmion/sorbitol to a 6h window as described above. The ring-stage parasites were diluted separately to 0.2% parasitemia (5% hematocrit) in the blood of three different donors. For the Nico-glmS cell line, the culture was split and glucosamine (Sigma # G1514) was added to one half of the culture (2.5 mM final concentration). The growth curve was performed in a 96-well plate (200 µl culture per well) with three technical replicates per condition and per blood. Parasitemia was measured every 24 h by staining parasite nuclei using SYBR Green I (Sigma # S9430). For the growth curve at 32°C and 37°C, the culture was split after synchronization, and additional timepoints were collected at the time of schizont rupture to precisely measure the duration of the life cycle at the different temperatures.
Instrument	CytoFLEX S cytometer (Beckman Coulter)
Software	FlowJo 10.10.0
Cell population abundance	Red blood cell (RBC) range: 16% - 87%<br>Singlet range of total RBCs indentified in the population: 98% - 99%<br>Infected RBCs (FITC+) range: 0.16% - 3.5%
Gating strategy	Red blood cells (RBC) were identified in FSC and SSC comparison. Singlets were gated using FSC(H) and FSC(A) of previously identified RBCs. P. falicparum infected RBC (iRBC) were selected inside the singlet (single) population using the FITC+ signal.

☒ Tick this box to confirm that a figure exemplifying the gating strategy is provided in the Supplementary Information.
