# Supplementary material for: A metabolism-chromatin axis promotes differential ribosomal RNA transcription in the human malaria parasite
Source: Nat Commun. 2025 Dec 19;17:818. doi: 10.1038/s41467-025-67522-w (PMC12824157; doi:10.1038/s41467-025-67522-w)
Supplement: Supplementary file 1 — Supplementary Information [file 41467_2025_67522_MOESM1_ESM.pdf]

A metabolism-chromatin axis promotes differential ribosomal RNA transcription in the human malaria parasite

Supplementary Figures 1-9

Supplementary Figure 1

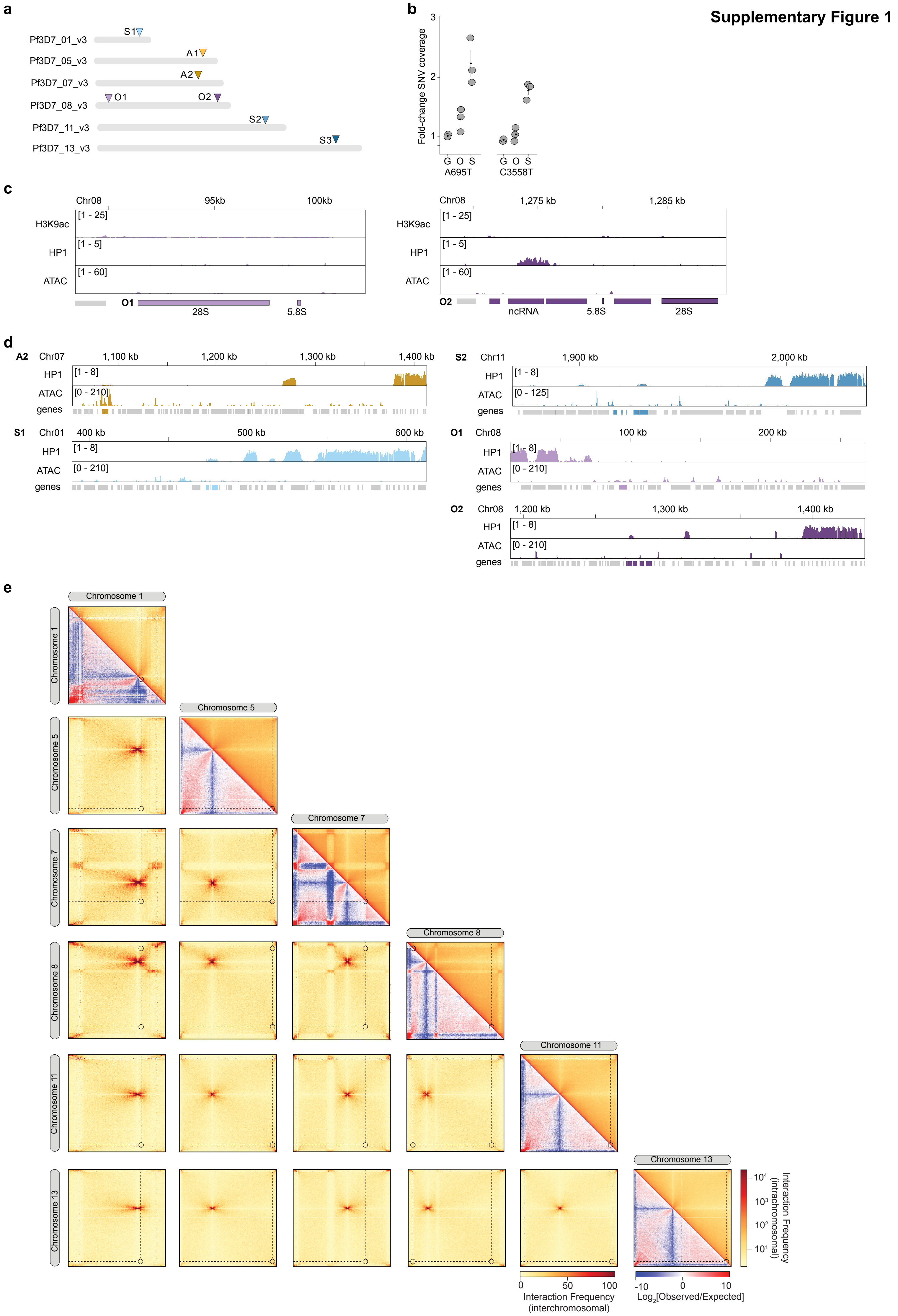

Supplementary Figure 1. **Organization of *P. falciparum* rDNA loci. a** Schematic representation of the localization of the seven rDNA loci in the *P. falciparum* genome. Only chromosomes with rDNA loci are represented. **b** Fold-change of the relative abundance of two A1 28S SNVs relative to the asexual stage in gametocytes (G), oocysts (O) and sporozoites (S). The SNV abundance can be calculated when reads originating from the masked A1 rDNA locus are mapped to the A2 rDNA locus. Black dot: mean; vertical lines: Standard error of the mean (SEM). n = 3 biological replicates. **c** H3K9ac and HP1 ChIP/input[1] (i.e. compacted, transcriptionally silenced chromatin) together with ATAC-seq21(assay for transposase accessible chromatin, i.e. open, transcriptionally active chromatin) ratio tracks at O-type rDNA loci. **d** HP1 ChIP/input ratio track[1] and ATAC-seq on the different rDNA loci[2]. The rDNA loci are colored in the genome track. **e** Micro-C derived intra- and interchromosomal interaction matrices of chromosomes containing a rDNA locus. All matrices are shown at 5 kb resolution, except intrachromosomal interactions of chromosome 1 (Pf3D7_01_v3, 2 kb). Interchromosomal and top triangle of the intrachromosomal matrices show interaction frequencies (see color bars). Bottom triangle of the intrachromosomal matrices show log_2-scaled observed/expected interaction frequency ratios.

Supplementary Figure 2

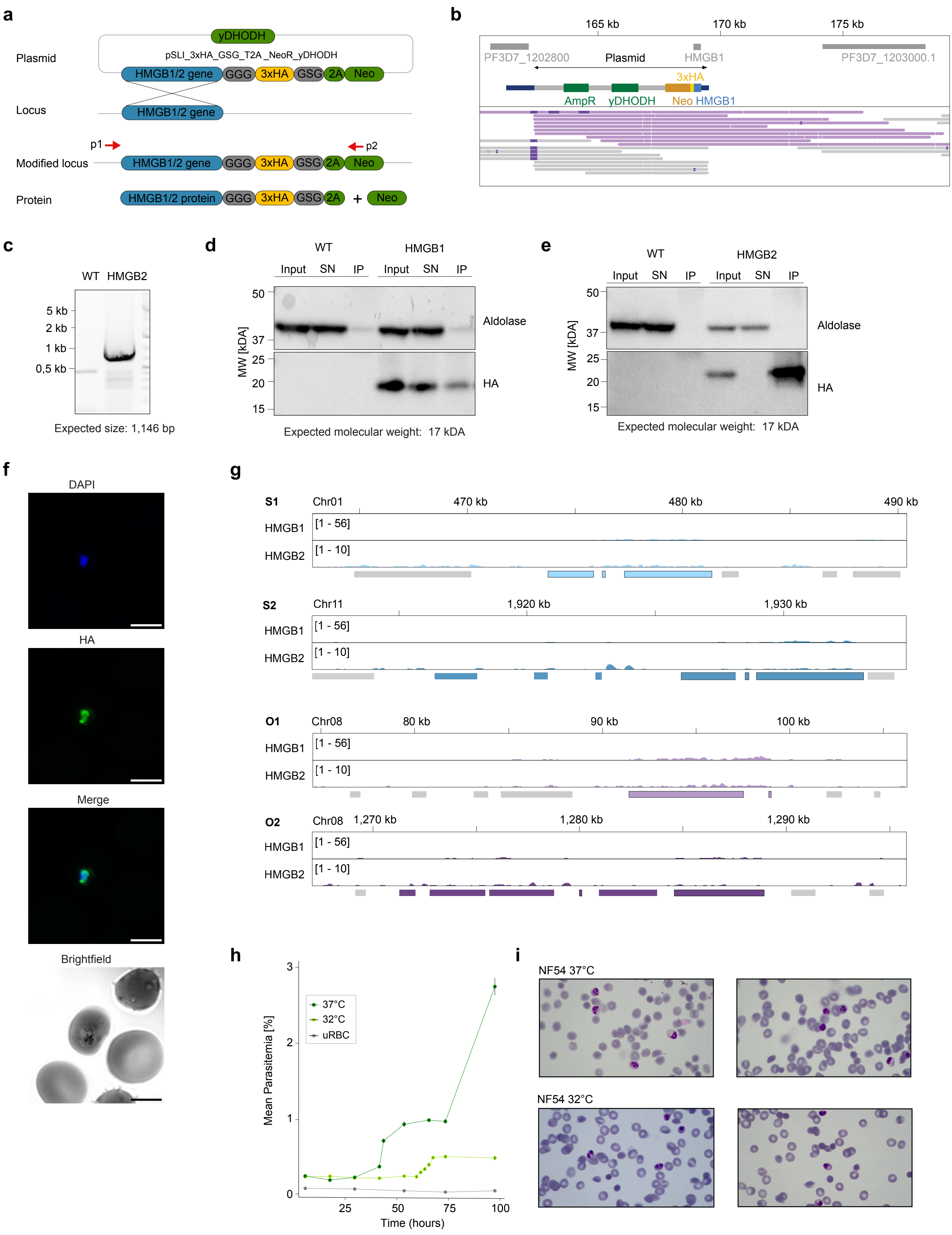

Supplementary Figure 2. **Validation of HMGB1/HMGB2 cell lines. a** Representation of the pSLI strategy to integrate a 3xHA-tag at the 3' end of the HMGB1 and HMGB2 loci. GGG and GSG: linkers; 2A: skip peptide; Neo: neomycin-resistance gene; yDHODH: yeast dihydroorotate dehydrogenase. **b** Nanopore tracks displaying reads (in purple) encompassing the pSLI plasmid, the HMGB1 locus and the surrounding genomic region, indicating correct integration of the plasmid downstream of the HMGB1 locus. **c** PCR of wild-type^{NF54} gDNA and HMGB2-HA gDNA using primer pair p1/p2 (Supplementary Fig. 2a, Supplementary Data 12) showing integration of the plasmid. **d** Western blot of a co-immunoprecipitation of HMGB1-HA performed with protein lysates extracted from wild-type parasites (negative control, left) and HMGB1-HA (right). Input: Input protein lysate; SN: Supernatant; IP: Immunoprecipitate. Aldolase is used as a loading control. Numbers on the left indicate molecular weight (MW) in kilodaltons (kDA). **e** As in D), but for HMGB2-HA. **f** Immunofluorescence assay of DNA (DAPI, blue) and HMGB1 (HA, green) showing a nuclear localization of the protein. **g** ChIP/input ratio tracks of HMGB1 and HMGB2 at the S1, S2, O1 and O2 loci at 37°C. **h** Growth curve showing parasitemia over 100 hours at 37°C and 32°C. Uninfected red blood cells (uRBC) served as background reference. Error bars: SEM of nine replicates (three independent blood donors x three technical replicates). **i** Giemsa images of wild-type^{NF54} parasites cultured at two different growth temperatures (37°C and 32°C) at the time of sampling for total RNA-seq (37°C: 36 h.p.i; 32°C: 56 h.p.i), showing a similar developmental stage.

Supplementary Figure 3

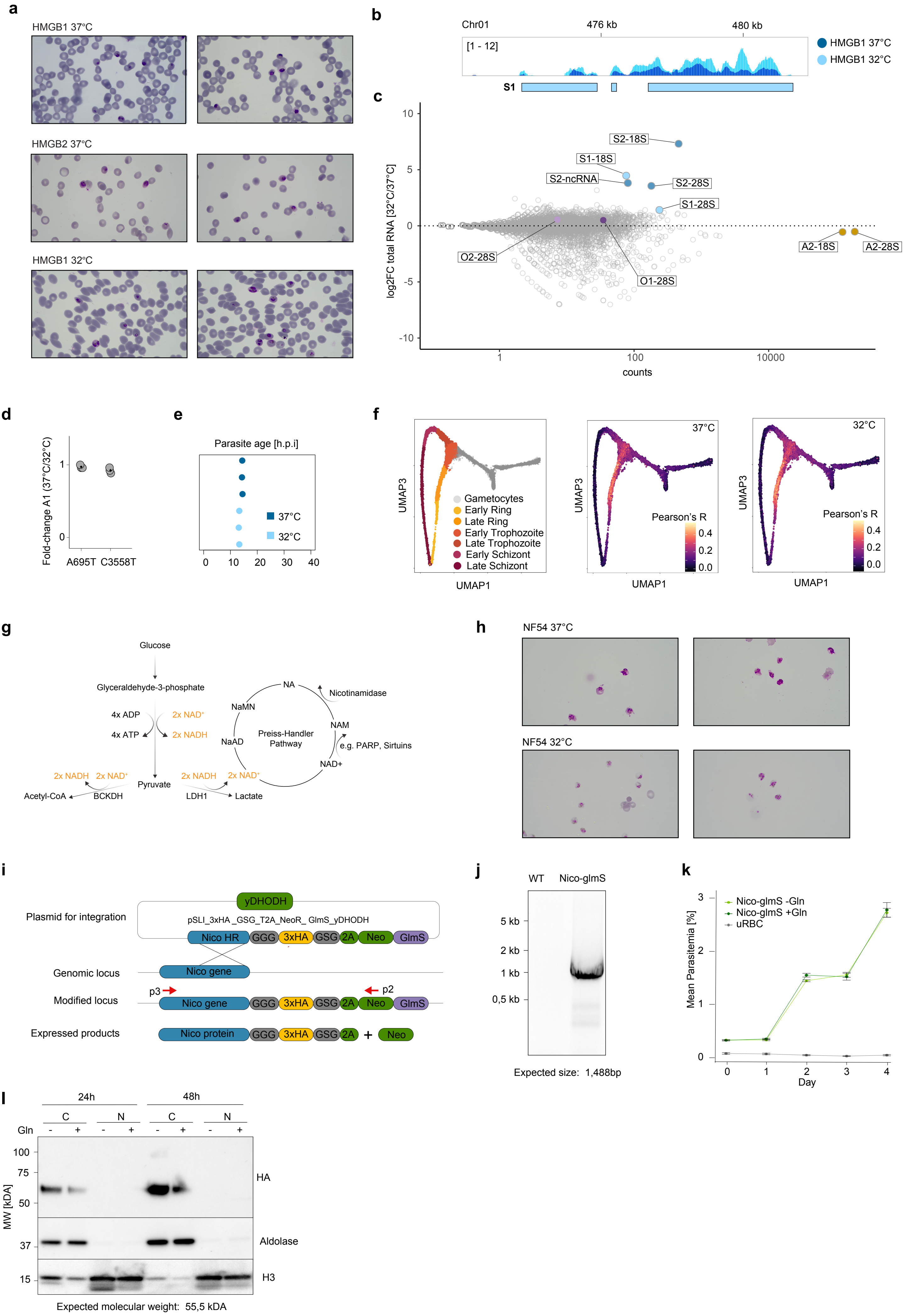

Supplementary Figure 3**. Comparison of parasite growth at different temperatures and validation of the Nico-glmS cell line. a** Giemsa images of HMGB1-HA and HMGB2-HA parasites cultured at 37°C (36 h.p.i.) and 32°C (56 h.p.i.) when sampled for ChIP-seq, showing similar developmental stages. **b** ChIP/input ratio track of HMGB1 at 32°C (light blue) and 37°C (dark blue) at the S1 rDNA locus. **c** Multidimensional scaling plot showing the changes of rRNA and ncRNA expression at 32°C compared to 37°C (total RNA-seq). **d** Fold-change of two A1 28S SNVs at 32°C relative to 37°C. Black dot: mean; vertical line: SEM. n = 3 biological replicates. **e** Developmental age of parasites grown at 37°C and 32°C derived from mRNA-seq vs a bulk reference transcriptome[3]. **f** Developmental age of parasites grown at 37°C and 32°C derived from mRNA-seq compared to scRNA reference (on the left[4]). **g** Schematic of NAD^+ consumption and regeneration during glycolysis ending either with Acetyl-CoA entering the TCA cycle (left) or lactate fermentation (right). NAD^+ can be used as a co-substrate and be regenerated in the Preiss-Handler Pathway. Numbers for NAD^+, NADH, ADP and ATP are per molecule of glucose. ADP: adenosine diphosphate; ATP: adenosine triphosphate; NAD^+/NADH: nicotinamide adenine dinucleotide, oxidized and reduced form; NAM: Nicotinamide; NA: nicotinic acid; NaMN: nicotinate mononucleotide; NaAD: nicotinate adenine dinucleotide; BCKDH: branched chain ketoacid dehydrogenase; LDH1: lactate dehydrogenase; PARP: poly (ADP-ribose) polymerases. **h** Giemsa images of wild-type^{NF54} parasites grown at 37°C and 32°C sampled for metabolomics experiments. **i** pSLI strategy to integrate a 3xHA-tag and a glmS ribozyme at the 3'end of the nicotinamidase locus. GGG and GSG: linkers; 2A: skip peptide; Neo: neomycin-resistance gene; yDHODH: yeast dihydroorotate dehydrogenase. **j** PCR of wild-type^{NF54} and Nico-glmS gDNA using primer pair p3/p2 (Supplementary Fig. 3i, Supplementary Data 12) showing plasmid integration. **k** Growth curve of the Nico-glmS cell line with (+Gln) and without (-Gln) glucosamine. uRBC served as background reference. Error bars: SEM of nine replicates (three independent blood donors x three technical replicates). **l** Western blot analysis of Nico-glmS parasites after 24/48h of glucosamine treatment. Aldolase and H3: loading controls of cytoplasmic (C) and nuclear (N) fractions . Numbers on the left indicate MW in kilodaltons.

Supplementary Figure 4

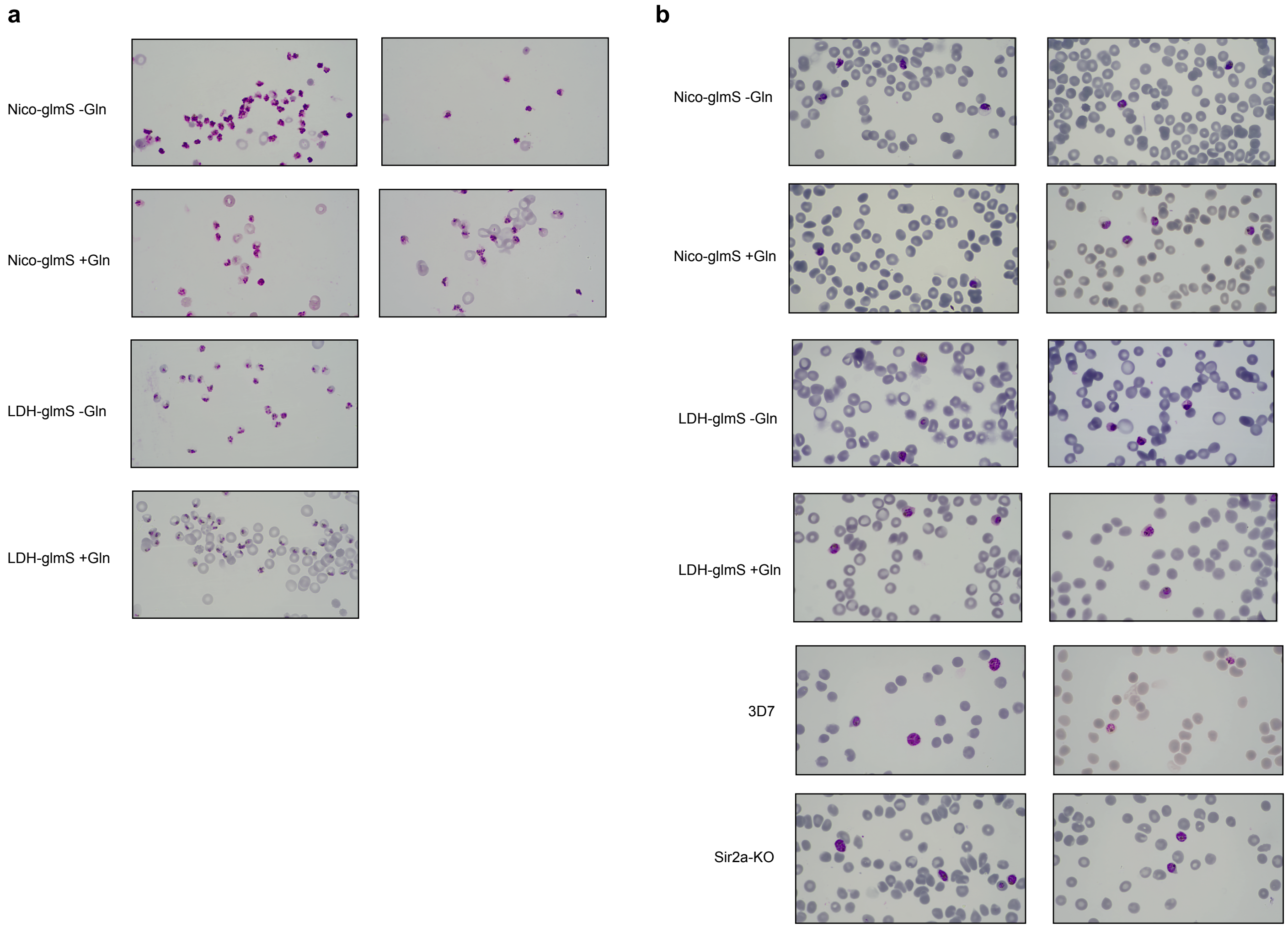

Supplementary Figure 4. **Giemsa images at the time of sampling for metabolomics and RNA-seq experiments. a** Giemsa images of indicated parasite cell lines and treatments at the time of sampling for metabolomics experiments. Gln: glucosamine. **b** Giemsa images of indicated parasite cell lines and treatments at the time of sampling for total RNA-seq experiments. Gln: glucosamine.

Supplementary Figure 5

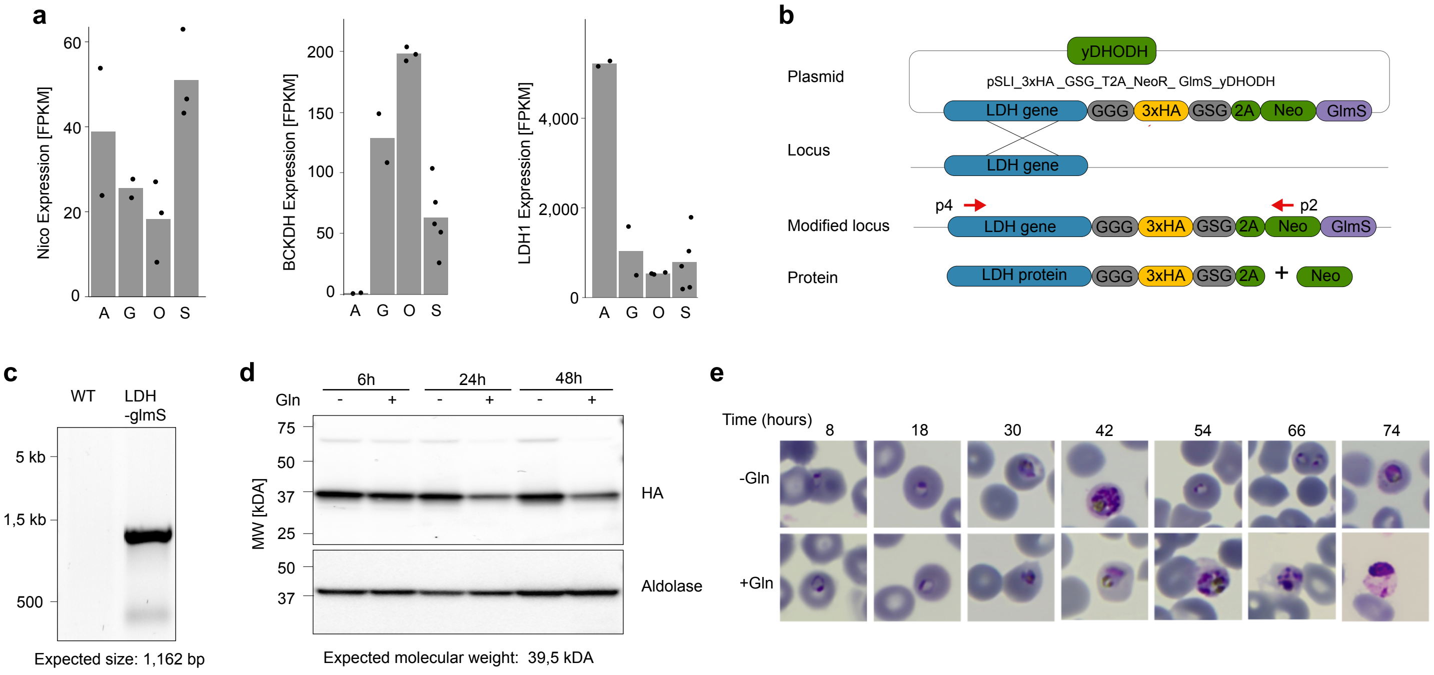

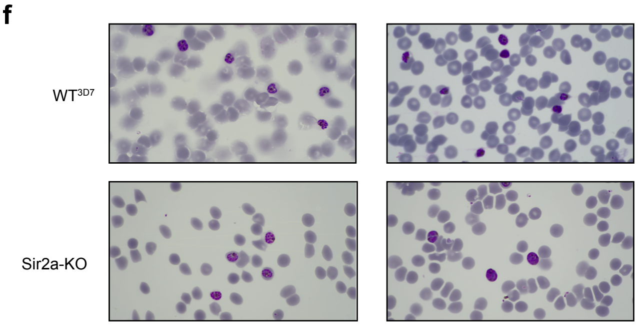

Supplementary Figure 5. **Expression profiles of Nico, BCKDH and LDH1 across the life cycle and LDH-glmS cell line validation. a** Expression of the nicotinamidase (Nico, PF3D7_0320500, left), branched chain ketoacid dehydrogenase (BCKDHA, PF3D7_1312600, middle) and lactate dehydrogenase (LDH1, PF3D7_1324900, right) along the parasite lifecyle. FPKM: fragments per kilobase of exon per one million mapped reads. A: asexual stages; G: gametocytes; O: oocysts; S: sporozoites. Data retrieved from[5,6]. Bar indicates the mean. n = 2 biological replicates for A and G; n = 3 biological replicates for O and S. **b** Representation of the pSLI strategy to integrate a 3xHA-tag and a glmS ribozyme at the 3'end of the LDH1 locus. GGG and GSG: linkers; 2A: skip peptide; Neo: neomycin-resistance gene; yDHODH: yeast dihydroorotate dehydrogenase. **c** PCR of wild-type^{NF54} and LDH-glmS gDNA using primer pair p4/p2 (Supplementary Fig. 5b, Supplementary Data 12) showing integration of the plasmid. **d** Western blot analysis of the LDH-glmS cell line after 6, 24 and 48h of glucosamine (Gln) treatment. Aldolase is used as a loading control. Numbers on the left indicate molecular weight (MW) in kilodaltons (kDA). **e** Giemsa images of LDH-glmS cells grown without (-Gln, top) or with (+Gln) glucosamine. Time represents hours after glucosamine additions. **f** Giemsa images of wild-type^{3D7} and Sir2a-KO parasites at the time of sampling for histone-PTM LC-MS/MS experiments.

Supplementary Figure 6

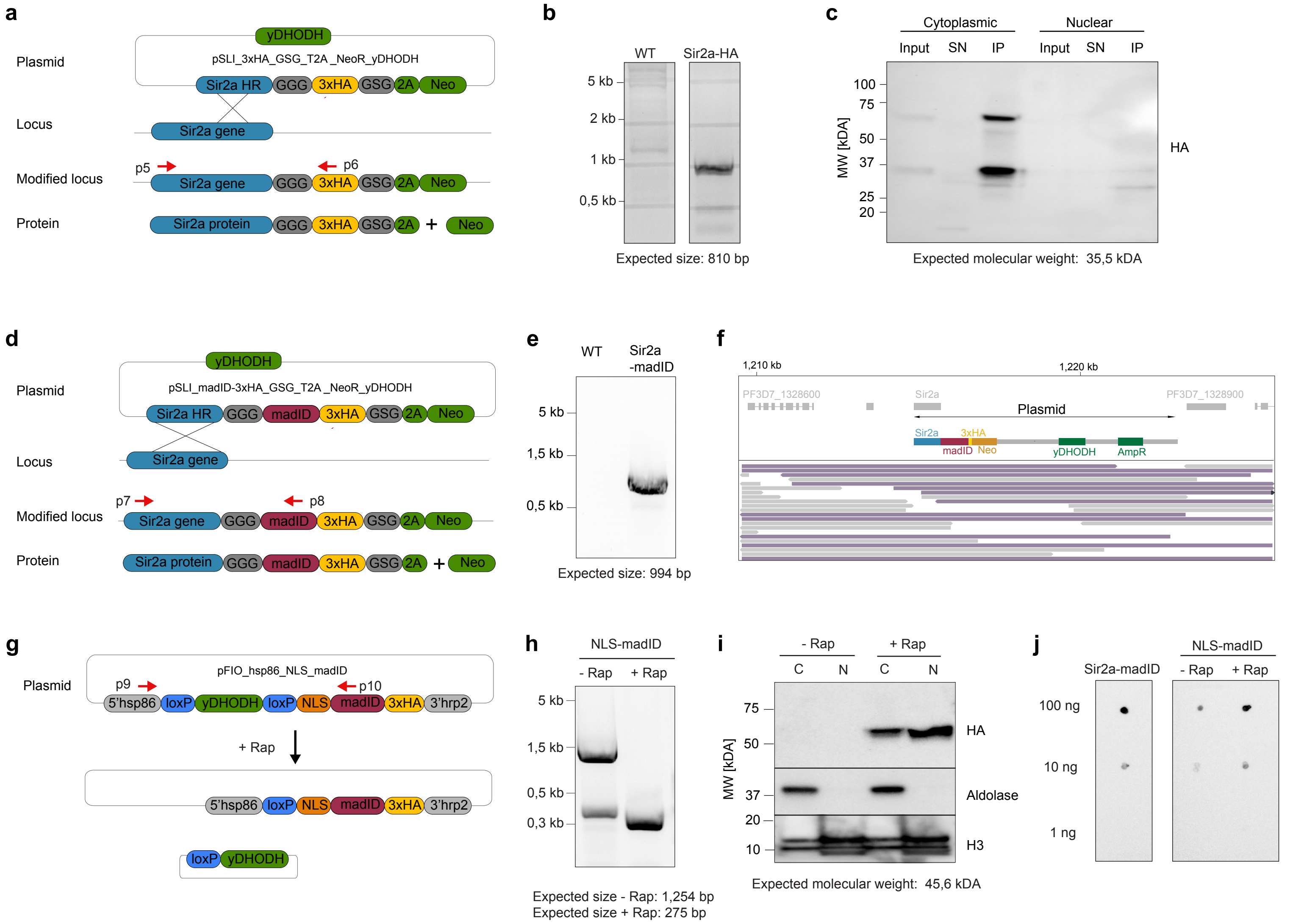

Supplementary Figure 6. **Validation of the 'madID' and 'Sir2a-madID' cell lines. a** Representation of the pSLI strategy to integrate a 3xHA-tag at the 3'end of the Sir2a locus. GGG and GSG: linkers; 2A: skip peptide; Neo: neomycin-resistance gene; yDHODH: yeast dihydroorotate dehydrogenase. **b** PCR of wild-type^{3D7} and Sir2a-HA gDNA using primer pair p5/p6 (Supplementary Fig. 6a, Supplementary Data 12) showing integration of the plasmid. **d** Western blot of a co-immunoprecipitation of Sir2a-HA performed with separated cytoplasmic (left) and nuclear protein extracts (right). Input: Input protein lysate; SN: Supernatant; IP: Immunoprecipitate. The higher band likely corresponds to a Sir2a-HA-neomycin fusion protein due to incomplete skipping at the T2A peptide site (calculated molecular weight of Sir2a-HA + neomycin: 64.4 kDA). Numbers on the left indicate MW in kilodaltons. **d** Representation of the pSLI strategy to integrate a madID-3xHA-tag at the 3'end of the Sir2a locus. GGG and GSG: linkers; 2A: skip peptide; Neo: neomycin-resistance gene; yDHODH: yeast dihydroorotate dehydrogenase. **e** PCR of wild-type^{3D7} and Sir2a-madID gDNA using primer pair p7/p8 (Supplementary Fig. 6d, Supplementary Data 12) showing integration of the plasmid. **f** Nanopore tracks displaying reads (in purple) encompassing both the pSLI plasmid, the Sir2a locus and the surrounding genomic region, indicating correct integration of the plasmid downstream of the Sir2a locus. **g** Representation of the pFIO_hsp86_NLS_madID plasmid used to inducibly overexpress the nuclear-localized madID protein under a hsp86 promoter. **h** PCR of NLS-madID gDNA without (-Rap, left) and with (+Rap, right) 200nM rapamycin treatment using primer pair p9/p10 (Supplementary Fig. 6g, Supplementary Data 12). yDHODH: yeast dihydroorotate dehydrogenase; NLS: nuclear localization signal; loxP: Cre recombinase binding sites. **i** Western blot analysis of the NLS-madID cell line without (-Rap, left) and with (+Rap, right) 200nM rapamycin treatment. Aldolase and H3 are used as loading controls for the cytoplasmic (C) and nuclear (N) fractions. Numbers on the left indicate molecular weight (MW) in kilodaltons (kDA). **j** Dot blot assay using anti-6mA antibodies against gDNA from Sir2a-madID cells (left) and NLS-madID cells without (-Rap, middle) and with (+Rap, right) 200 nM rapamycin treatment. Numbers on the left indicate total amount of gDNA used.

Supplementary Figure 7

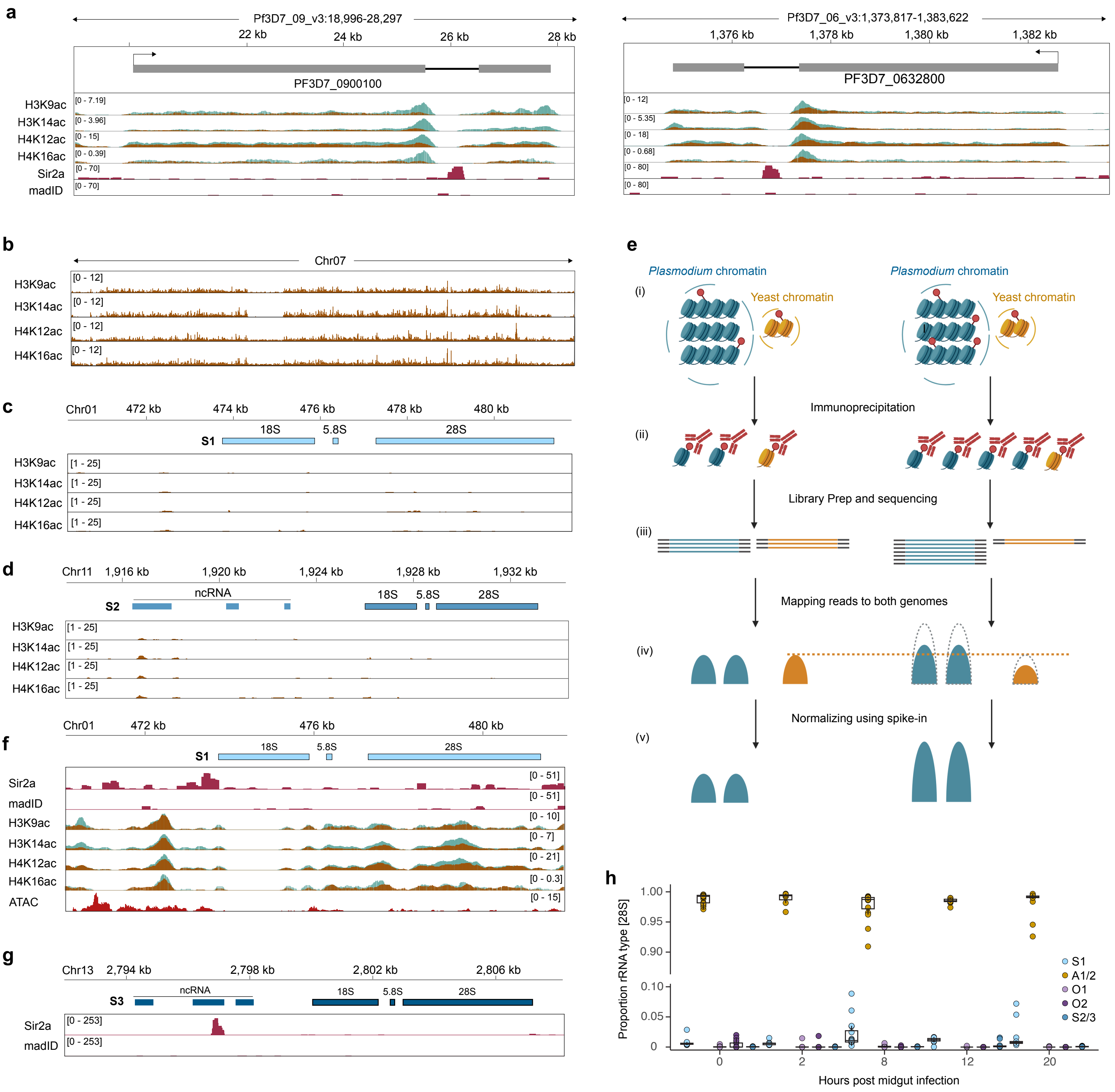

Supplementary Figure 7. **Histone PTMs and Sir2a binding at subtelomeric var and rDNA loci.** **a** Detailed view of calibrated ChIP-seq data for four histone PTMs at two subtelomeric *var* genes. Blue: Sir2a-KO; brown: Wild-type[3D7]. Sir2a-madID ('Sir2a') and NLS-madID ('madID') are shown as number of modified sites per 10 bp bin normalized to the AT content of each bin. **b** ChIP/input track of the second replicates for H3K9ac, H3K14ac, H4K12ac and H4K16ac on chromosome 7. **c** ChIP/input ratio tracks of H3K9ac, H3K14ac, H4K12ac and H4K16ac at the S1 rDNA locus. **d** ChIP/input ratio tracks of H3K9ac, H3K14ac, H4K12ac and H4K16ac at the S2 rDNA locus. **e** Illustration representing the principle of calibrated ChIP-seq using spike-in chromatin[7]. Yeast and *P. falciparum* chromatin are crosslinked and sonicated separately. A known quantity of yeast chromatin is added to each *P. falciparum* sample (i) and the common ChIP-seq protocol is followed (ii). Following library preparation and sequencing (iii), the reads are aligned in parallel to the genome of interest (here *P. falciparum*) and the 'calibration' genome from which the spike-in sample is derived (here *S. cerevisiae*). Given that the amount of spike-in chromatin is known, the number of reads mapping to the 'calibration genome' in each sample, replication and condition (iv) can then be used to normalize the ChIP signal in the *P. falciparum* genome (v) (see Methods). Created in BioRender. Baumgarten, S. (2026) https://BioRender.com/u6l6q32. **f** Detailed view of 6mA densities in Sir2a-madID ('Sir2a'), NLS-madID ('madID') and calibrated ChIP data for four histone PTMs at the S1 rDNA locus. Blue: Sir2a-KO; brown: wild-type[3D7]. madID data are shown as number of modified sites per 10 bp normalized to the AT content of each bin. **g** Detailed view of 6mA densities in Sir2a-madID ('Sir2a'), NLS-madID ('madID') at the S3 rDNA locus. **h** Proportion of A1/2, S1, S2/3, O1 and O2 28S rRNA during midgut development of *P. falciparum*. Time indicates hours post bloodmeal. Boxes represent the median and interquartile range (IQR). Whiskers represent 1.5x IQR. Single-cell RNA-seq data were re-analyzed from[8] (see Methods). n (i.e. individual cells) = 16 (T0), 7 (T2), 16 (T8), 12 (T12) and 13 (T20).

Supplementary Figure 8

RBCs gating

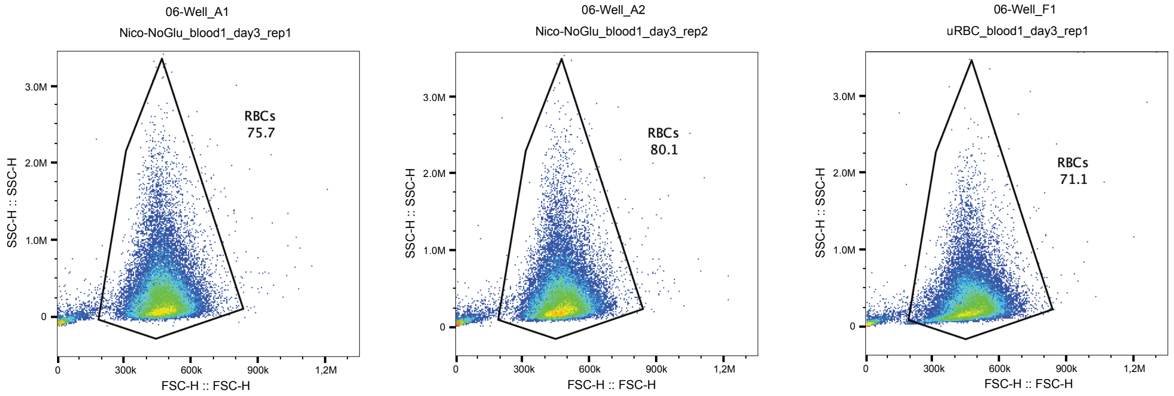

Singlets gating

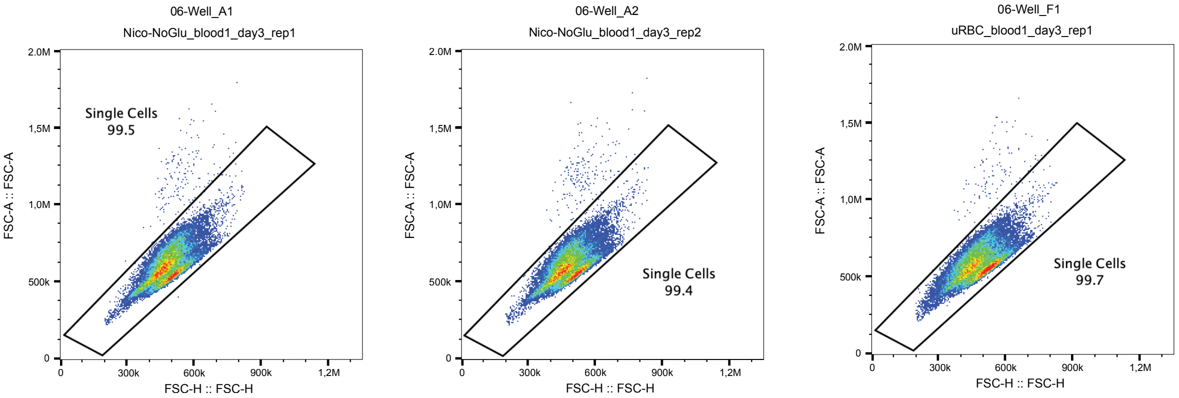

FITC+ gating

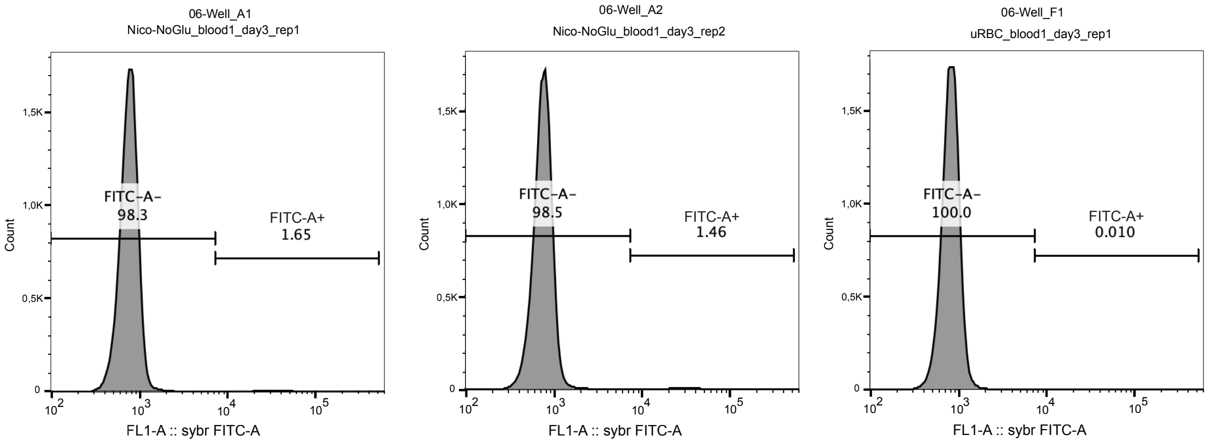

Sample Name	Events	RBCs	Single Cells	FITC+
06-Well-A1	39539	29941	29792	491
06-Well-A2	37381	29948	29765	436
06-Well-F1	42312	30073	29970	3

Supplementary Figure 8. **Exemplary overview of the gating strategy used to measure parasite growth.**

Supplementary Figure 9

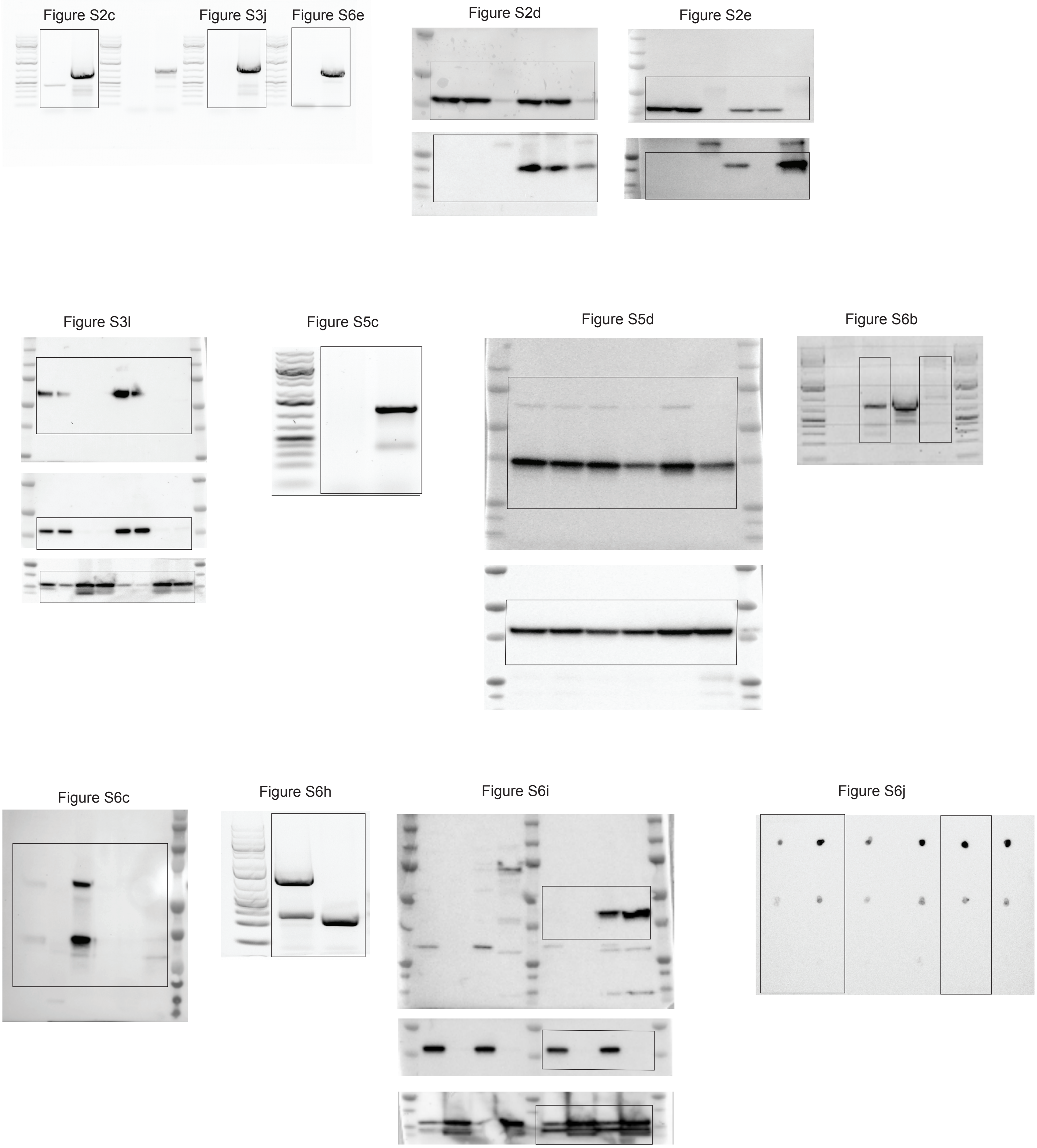

Supplementary Figure 9. **Uncropped images of DNA gels and Western Blot membranes used in this study.**

References

1. Carrington, E. *et al*. The ApiAP2 factor PfAP2-HC is an integral component of heterochromatin in the malaria parasite *Plasmodium falciparum. iScience* **24**, (2021).
2. Toenhake, C. G. et al. Chromatin Accessibility-Based Characterization of the Gene Regulatory Network Underlying *Plasmodium falciparum* Blood-Stage Development. *Cell Host Microbe* **23**, 557-569.e9 (2018).
3. Lemieux, J. E. *et al*. Statistical estimation of cell-cycle progression and lineage commitment in *Plasmodium falciparum* reveals a homogeneous pattern of transcription in ex vivo culture. *Proc Natl Acad Sci USA* **106**, 7559–64 (2009).
4. Dogga, S. K. *et al*. A single cell atlas of sexual development in *Plasmodium falciparum*. *Science* **384**, (2024).
5. Zanghì, G. *et al*. A Specific PfEMP1 Is Expressed in *P. falciparum* Sporozoites and Plays a Role in Hepatocyte Infection. *Cell Rep* **22**, 2951–2963 (2018).
6. Baumgarten, S. *et al*. Transcriptome-wide dynamics of extensive m6A mRNA methylation during Plasmodium falciparum blood-stage development. *Nat Microbiol* **4**, 2246–2259 (2019).
7. Orlando, D. A. *et al*. Quantitative ChIP-Seq normalization reveals global modulation of the epigenome. *Cell Rep* **9**, 1163–70 (2014).
8. Mohammed, M. *et al*. Single-Cell Transcriptomics To Define *Plasmodium falciparum* Stage Transition in the Mosquito Midgut. *Microbiol Spectr* **11**, e0367122 (2023).
